# A Comparative Study of Relational Learning Capacity in Honeybees (*Apis mellifera*) and Stingless Bees (*Melipona rufiventris*)

**DOI:** 10.1371/journal.pone.0051467

**Published:** 2012-12-10

**Authors:** Antonio Mauricio Moreno, Deisy das Graças de Souza, Judith Reinhard

**Affiliations:** 1 Departamento de Psicologia, Universidade Federal de São Carlos, São Carlos, São Paulo, Brazil; 2 National Institute of Science and Technology on Behavior, Cognition, and Teaching, Universidade Federal de São Carlos, São Carlos, São Paulo, Brazil; 3 Queensland Brain Institute, The University of Queensland, Brisbane, Queensland, Australia; Royal Holloway University of London, United Kingdom

## Abstract

**Background:**

Learning of arbitrary relations is the capacity to acquire knowledge about associations between events or stimuli that do not share any similarities, and use this knowledge to make behavioural choices. This capacity is well documented in humans and vertebrates, and there is some evidence it exists in the honeybee (*Apis mellifera*). However, little is known about whether the ability for relational learning extends to other invertebrates, although many insects have been shown to possess excellent learning capacities in spite of their small brains.

**Methodology/Principal Findings:**

Using a symbolic matching-to-sample procedure, we show that the honeybee *Apis mellifera* rapidly learns arbitrary relations between colours and patterns, reaching 68.2% correct choice for pattern-colour relations and 73.3% for colour-pattern relations. However, *Apis mellifera* does not transfer this knowledge to the symmetrical relations when the stimulus order is reversed. A second bee species, the stingless bee *Melipona rufiventris* from Brazil, seems unable to learn the same arbitrary relations between colours and patterns, although it exhibits excellent discrimination learning.

**Conclusions/Significance:**

Our results confirm that the capacity for learning arbitrary relations is not limited to vertebrates, but even insects with small brains can perform this learning task. Interestingly, it seems to be a species-specific ability. The disparity in relational learning performance between the two bee species we tested may be linked to their specific foraging and recruitment strategies, which evolved in adaptation to different environments.

## Introduction

Relational learning defines the capacity to acquire knowledge about the relations, associations, and interactions between different stimuli, objects or events. Matching-to-sample procedures [1] are typically used for investigating relational learning. When presented with a sample stimulus, a human or non-human subject can be taught to select, from an array of comparison stimuli, a second stimulus identical to this sample (*identity matching-to-sample*), or to select the stimulus that differs from the sample (*oddity matching-to-sample*). The subject can also learn to associate stimuli that do not share physical similarities (*arbitrary or symbolic matching-to-sample*), for example selecting a yellow stimulus when presented with a sample stimulus consisting of black and white vertical patterns and selecting a blue stimulus when presented with a sample stimulus consisting of black and white horizontal patterns.

It has long been known that bees have an impressive capacity to learn and discriminate colours, shapes, patterns and odours [2]–[6]. These learning abilities have been investigated mostly in the common honeybee *Apis mellifera*, but also in bumblebees [7]–[12], and even new-world stingless bees, such as *Melipona rufiventris* and *M. quadrifasciata* [13]–[17]. Using Y-maze set-ups, where bees are presented with a sample stimulus followed by two choice stimuli, Giurfa et al. [18] demonstrated that *Apis mellifera* can learn matching-to-sample and non-matching-to-sample relations between visual stimuli such as colours. They further showed that *Apis mellifera* is also able to transfer the concepts of “sameness” and “difference” from colours to novel stimuli such as patterns and vice versa, and even cross-modally from odours to colours [18]. Prior to this study, such a level of learning performance was demonstrated only in vertebrates.

Honeybees can also learn arbitrary relations between stimuli: symbolic matching-to-sample procedures have been used to train *Apis mellifera* to associate patterns with colours, and vice versa [19], [20]. In the study by Zhang et al. [19], bees learnt the relations between three visual stimuli (grating orientation, colour, and pattern) that were presented in a specific order. When the stimulus sequence was changed, i.e. the stimuli were presented in a different order, the bees still showed a significant percentage of correct choices, which could be interpreted as demonstration that bees had learnt emergent symmetries between the stimuli. However, in the study by Zhang et al. [19] correct responses were always rewarded even when stimulus sequences were reversed. Therefore, the bees might have merely learnt new relations between the stimuli in a different order. To investigate whether honeybees truly have the capacity for learning emergent symmetries within stimulus relations, the stimuli need to be presented without positive reinforcement in a reversal test.

While *Apis mellifera’s* capacity for learning symmetrical relations remains to be investigated, it is known that its relational learning ability extends beyond patterns and colours. Honeybees can learn cross-modal arbitrary relations, i.e. associating stimuli from different sensory modalities with each other, such as lemon scent with the colour blue, and mango scent with the colour yellow [21]. This capacity for cross-modal associations was further confirmed by field experiments, in which honeybees learnt to associate scents with different feeder colours, and returned to the coloured feeders whenever the corresponding scent was blown into the hive, irrespective of the feeder location [22].

Stingless *Melipona* bees are known to have considerable associative learning capacities both in the visual and the olfactory domain [14]–[17], but their capacity for relational or other complex forms of learning has not been well investigated. One early study explored relational learning of light intensities and colours in *Melipona rufiventris* [23]. Here, bees had to learn the relation between the light condition of a chamber (light on or off), and the colour around the feeder. However, due to the design of the experiment it could not be shown conclusively whether *Melipona* learnt the relations between the stimuli or merely stimulus configurations. In the present study, we therefore investigated the capacity for learning arbitrary relations between colours and patterns in *Melipona rufiventris*, in direct comparison to the relational and symmetrical learning abilities of *Apis mellifera*, demonstrating existence of this capacity in one species, but not the other.

## Results

### Learning of Arbitrary Relations in *Apis mellifera*

**Figure 1:**
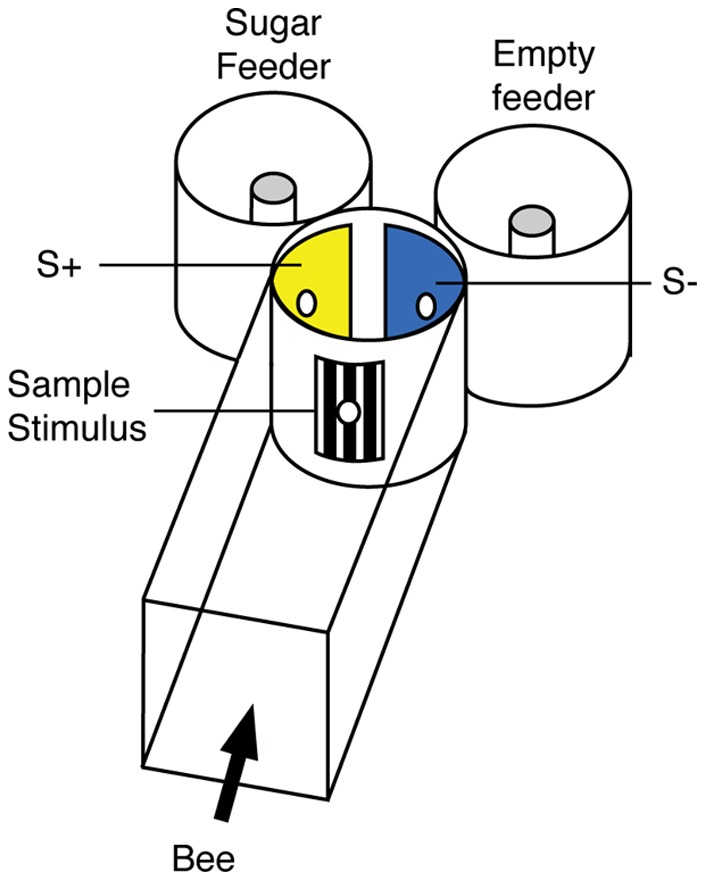
Schematic of the Y-maze apparatus used for investigating learning of arbitrary relations in honeybees. Bees entered a tunnel, flew through the maze entrance (marked with the sample stimulus) and then flew through one of two exits (each marked with a comparison stimulus). A correct choice (S+) led to a feeder containing sugar solution. An incorrect choice (S–) led to an empty feeder.

**Figure 2:**
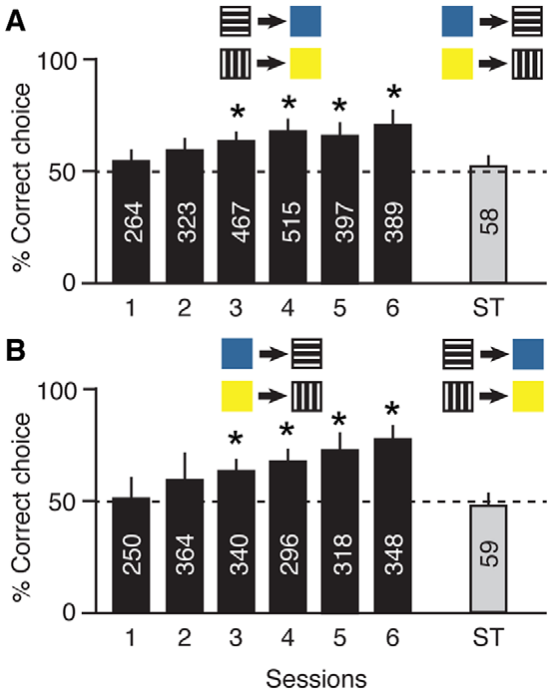
Learning of arbitrary relations in *Apis mellifera* honeybees. Black bars: mean percentage (± s.d.) of correct choices in six rewarded training sessions of seven training blocks each, during which one group of 20 bees learnt the relations patterns-colours (A), and a second group of 20 bees learnt the relations colours-patterns (B). Grey bar: mean percentage (± s.d.) of correct choices in an unrewarded symmetry test session (ST) during which the bees were presented with the stimuli in reverse order to the training sessions. Numbers inside bars indicate the total number of choices per session. Asterisks above columns indicate sessions in which the mean number of correct choices was significantly different to chance (50%) at *P*<0.001.

We trained 20 individually marked *Apis mellifera* workers to navigate a Y-maze (Fig. 1), where they first encountered a sample stimulus (black and white pattern, either horizontal or vertical) and then two comparison stimuli (the colours blue and yellow). The bees had to learn the arbitrary relation between horizontal pattern and the colour blue, and vertical pattern and the colour yellow. They received a sugar reward when choosing the correct comparison stimulus. A second group of 20 individual *A. mellifera* bees were trained in the reverse sequence, namely relating a blue sample stimulus to a horizontal pattern, and a yellow sample stimulus to a vertical pattern. The bees were trained in this way in six sessions, each consisting of seven 30-minute training blocks, over six consecutive days (for details of the procedure see Materials and Methods). The bees’ choices were pooled per block for each session. *A. mellifera* honeybees easily learnt the arbitrary pattern-colour (Fig. 2A) and colour-pattern (Fig. 2B) relations. Learning performance, measured as percentage of correct choices per session, significantly increased over the sessions from 56.2% to 68.2% for pattern-colour relations (F_5,30_ = 7.91, *P*<0.001), and from 51.5% to 73.3% for colour-pattern relations (F_5,30_ = 13.71, *P*<0.001).

After having learnt these arbitrary relations the bees were then tested for emergent symmetrical relations between the training stimuli, where the respective stimuli were presented in reverse order to the training, and the bees’ choices were unrewarded. That is, the stimuli changed their function with sample stimuli becoming comparison stimuli and vice versa. The bees that were trained to relate patterns to colours were tested with the corresponding colour-pattern combinations, and bees trained to relate colours to patterns were tested with the corresponding pattern-colour combinations (Fig. 2). In spite of successfully learning arbitrary relations, *Apis mellifera* bees were not able to transfer this knowledge to the symmetry test. The mean percentage of correct responses with symmetrical relations was 51.7% (Fig. 2A) and 47.5% (Fig. 2B), respectively, both results not differing statistically from chance (one-sample t-test, *P* = 0.307 and *P* = 0.548).

### Learning of Arbitrary Relations in *Melipona rufiventris*

**Figure 3:**
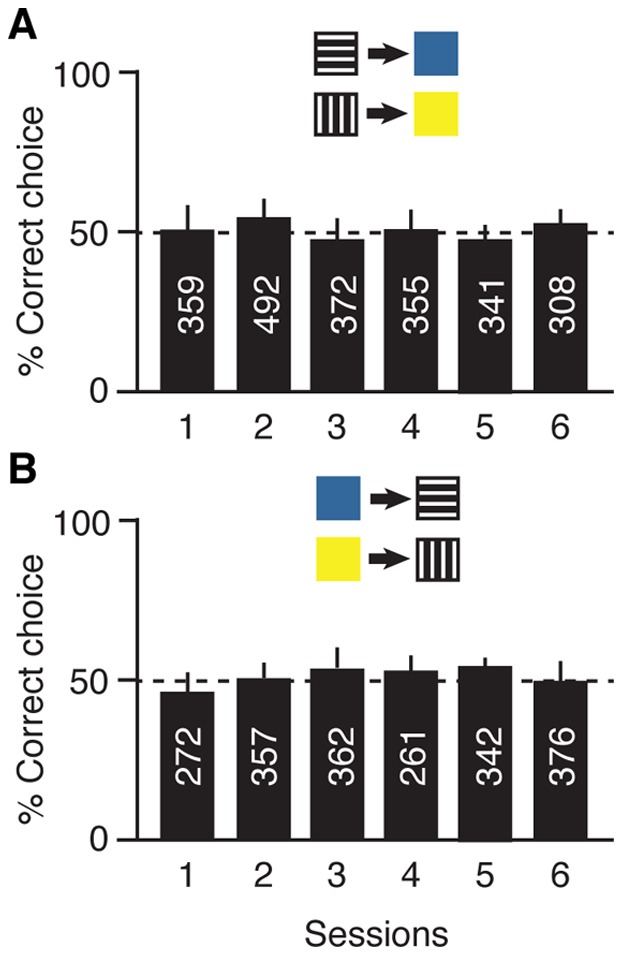
Learning of arbitrary relations in *Melipona rufiventris* stingless bees. Bars: mean percentage (± s.d.) of correct choices in six rewarded training sessions of seven training blocks each, during which one group of 8 bees learnt the relations patterns-colours (A), and a second group of 8 bees learnt the relations colours-patterns (B). Numbers inside bars indicate the total number of choices per session.

We repeated the experiment on learning of arbitrary relations with *Melipona rufiventris*, a stingless honeybee from Brazil. Due to the difficulties in training this species compared to *Apis* (see Materials and Methods), only eight individual *M. rufiventris* workers were used per group. As for *Apis,* one group was trained to relate patterns to colours, and the second group trained to relate colours to patterns, in six sessions over six consecutive days with each session consisting of seven 30-minute training blocks. Although fewer individuals were used with *Melipona*, the overall number of choices per session was in a similar range to *Apis*, because the experimental apparatus was installed right next to the *Melipona* colony allowing more frequent return visits per session. In spite of the extensive training, *M. ruvifentris* workers did not learn the arbitrary relations between patterns and colours (Fig. 3A), or colours and patterns (Fig. 3B). Learning performance did not increase over the six sessions for pattern-colour relations (F_5,30_ = 0.745, *P* = 0.596), nor for colour-pattern relations (F_5,30_ = 0.71, *P* = 0.62), remaining at chance levels.

**Figure 4:**
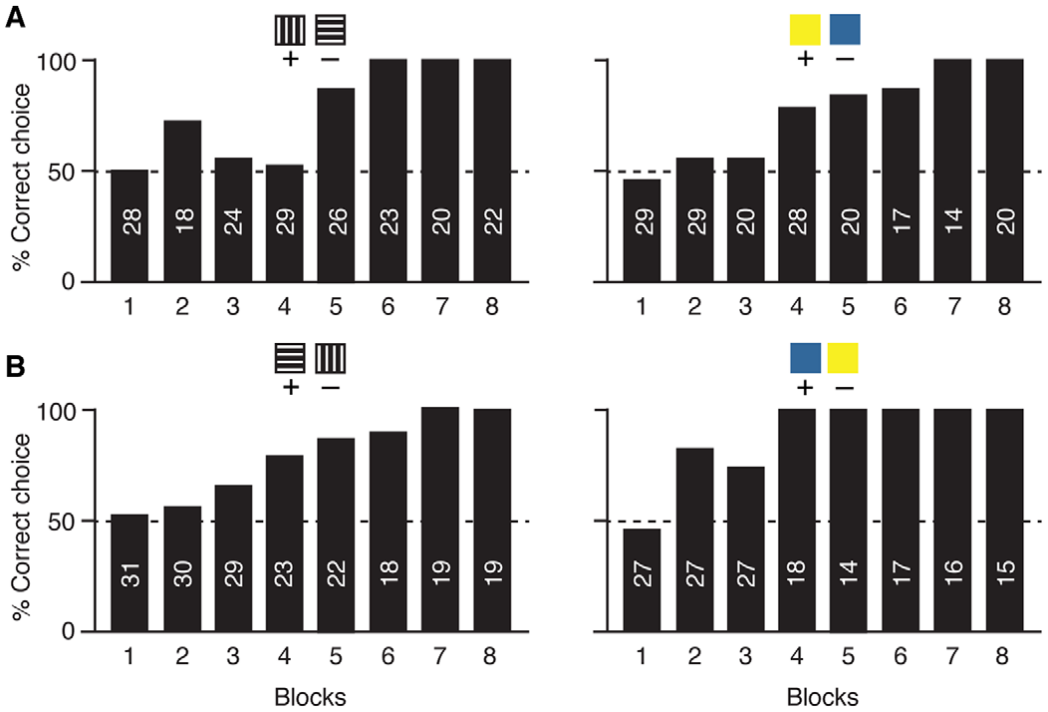
Discrimination learning in *Melipona rufiventris* stingless bees. Bars: percentage of correct choices in eight consecutive training blocks, during which bees learnt to discriminate a vertical pattern from a horizontal pattern, and yellow from blue. One group of 8 bees was trained with vertical and yellow as rewarded stimuli (A), and a second group of 8 bees was trained with horizontal and blue as rewarded stimuli (B). The learning criterion was 100% correct choices in at least two consecutive blocks. Numbers inside bars indicate the total number of choices per session.

Due to their inability to learn arbitrary relations, *M. rufiventris* bees were not tested for the symmetrical relations. Instead, we investigated whether their poor learning performance could be due to an inability to discriminate the pattern and colour stimuli. Therefore, the 16 *M. rufiventris* bees received simple discrimination training with horizontal vs vertical patterns and blue vs yellow colours, with one group of 8 bees trained with vertical and yellow as rewarded stimuli (Fig. 4A), and the second group of 8 bees trained with horizontal and blue as rewarded stimuli (Fig. 4B). Each bee received eight consecutive 15-minute training blocks until 100% correct responses in at least two consecutive blocks was reached. All bees learnt to discriminate patterns as well as colours rapidly, achieving the learning criterion of 100% correct choice after 4, 6 or 7 blocks, respectively (Fig. 4).

## Discussion

Our study showed that *Apis mellifera* is capable of learning arbitrary relations between stimuli, confirming previous findings revealing that this small insect has impressive cognitive capacities [18]–[22], [24], [25]. However, *Apis mellifera* was apparently unable to use this relational information when tested with symmetrical relations, where the stimulus order was reversed. These findings contrast with earlier results, where *Apis mellifera* workers learnt the relations between three visual stimuli (gratings A vs A’, colours B vs B’, patterns C vs C’) [19]. In this earlier study, bees learnt the sequences ABC and A’B’C’, with learning performance equivalent to our study. When presented with different stimulus sequences, e.g. BAC vs B’A’C’ or CBA vs C’B’A’, the bees still showed a significant percentage of correct choices, which could be interpreted as a demonstration of emergent symmetry. However, in this study all sessions were conducted under reinforcement conditions; that is responses to the correct stimuli were always rewarded even during sequence reversal [19]. It is therefore conceivable that the bees in the Zhang et al [19] study did not demonstrate emergent symmetry, but merely learnt the new relations between the stimuli expedited by the fact that they were already familiar with one relationship between the stimuli. In contrast, our study presented the stimuli without positive reinforcement during the stimulus reversal, and is thus a true test for symmetrical relations.

Our results seem to suggest that *Apis mellifera* do not learn stimulus relations in a symmetrical fashion, in line with their inability to establish transitive inferences [26]. When presented with multiple stimulus relations, bees guide their choices by recency effects combined with associative strength of the stimuli [26]. In our study the comparison stimuli were directly associated to the reward, while the sample stimulus may function as a navigational ‘landmark’ for the bees and therefore have lower associative strength. When the stimuli were reversed in the symmetry test, the bees might only pay attention to the sample stimulus, which previously was directly associated with the sugar reward, and consider the comparison stimuli as not immediately relevant for obtaining the food. This would result in random choice, as observed in our test.

In contrast to honeybees, *Melipona rufiventris* seems incapable of learning the arbitrary relations of patterns and colours tested here, and hence were not tested for symmetrical relations. *M. rufiventris’* apparent inability for relational learning is not due to sensory limitations as they are clearly able to discriminate between the stimuli used. Nor is it due to a general inability or inadequacy to learn: associative learning in *Melipona* bees has been shown both for the visual and olfactory domain [13]–[17], and *Melipona rufiventris* is even capable of operant learning [27]. Recent preliminary work from our laboratory using a modified matching-to-sample procedure in a T-maze indicate similar results to the findings presented here: both *M. rufiventris* and *M. quadrifasciata* seem to acquire identity relations very quickly, but fail to learn arbitrary relations involving visual stimuli as well as olfactory stimuli (Moreno and de Souza, pers. comm.). There is one study suggesting *M. rufiventris* can learn relations between colours and light intensities [23]. However, because both stimuli were presented simultaneously in that study, and not successively (as is the case in matching-to-sample procedures), it is possible that the bees had learnt stimuli configurations instead of relations [28]. Nevertheless, it is clear that *Melipona* species have good learning abilities. Why then are they not able to learn arbitrary relations?

An animal’s learning abilities presumably have evolved to suit the lifestyle within its ecological niche, and species-specific variations in learning performance are thought to be an adaptation to the particular environment in which the animal operates [29]. *Melipona*’s inability to learn arbitrary relations may be intrinsically linked to its foraging biology. *Melipona* bees including *M. rufiventris* have much narrower food niches than *Apis mellifera;* the spectrum of plants visited by *Melipona* bees is rather restricted, and they are less generalistic than *Apis* in terms of flower choice [30]–[33]. *Melipona* focuses on mass-flowering trees that produce a burst of dense inflorescence clusters for a short period of time. Due to their small colonies of less than 1000 individuals *Melipona* species do not require numerous different food sources that are available at the same time to sustain themselves [34]. Their narrower foraging niche may reduce the need to simultaneously learn multiple visual cues from a broad spectrum of flower types that are distributed over a large foraging area, as *Apis mellifera* does. It is conceivable that under the latter conditions flexibility in learning food-related cues is more important, and may have driven the evolution of relational learning in *A. mellifera*. Of course, this argument is hypothetical at this stage and requires the support of detailed comparative studies of the food sources and visual stimuli that each species uses during foraging – from flower cues to navigational landmarks. Such studies would have to take into account the specific foraging environment a particular colony and individual foragers experience. For example, an *A. mellifera* colony might exploit a larger variety of food sources than an *M. rufiventris* colony in general; but depending on the particular colony site, individual *A. mellifera* bees may be exposed to either rapidly changing food sources (and hence an individual would have to learn a variety of different flowers in its life time), or bees might have access to food sources that flower for extended periods (and hence individual bees would only need to learn one or two flower types in their life time).

Another aspect that might contribute to the difference in relational learning capacity between *Apis* and *Melipona*, is their differences in recruitment strategies. *Melipona* species use comparatively simple communication strategies to recruit nest-mates to food sources. Although there are a diversity of species-specific recruitment mechanisms in meliponine bees, such as jostling, spinning, body contacts and sound production inside the nest, visual guidance flights, odour trails and foot-print marks on food sources [35]–[37], none of these mechanisms reach the sophistication and complexity of the honeybee dance language produced exclusively by bees of the genus *Apis* [3], [38]. The simpler recruitment and foraging mechanisms in *Melipona*, and the lack of a complex dance language might have prevented the evolution of more “abstract” learning abilities in stingless bees such as required for learning of arbitrary relations.

Species-specific differences in learning performance have been shown in many insect groups including bees, parasitoid wasps, and butterflies [29], [39]–[42]. However, natural within-species variations can affect between-species comparisons. Significant intraspecific differences in learning performance depending on the population or colony have been shown for both honeybees and bumblebees [39], [43]–[45]. It has to be taken into account that our study was carried out with only one colony per species and a rather limited number of *Melipona* individuals. To provide conclusive evidence that the observed differences in relational learning performance between *A. mellifera* and *M. rufiventris* are indeed species-specific, the results would need to be confirmed with more colonies.

Furthermore, the study was carried out in two different countries due to the fact that *Melipona rufiventris* is native to Brazil and cannot be introduced into Australia for ecological reasons. Although the experimental apparatus and protocol were the same, and we tried to keep the settings as similar as possible, different weather conditions, differences in food availability and colony health could potentially have contributed to the species differences in learning performance. There is also the possibility that our experimental set-up may not have been suitable for demonstrating arbitrary learning in *Melipona rufiventris*. Indeed, the fact that we kept experimental conditions identical could have been a disadvantage. Finding differences in learning between two species that were tested under the same experimental conditions might reflect true species differences or, alternatively, may reflect differences in how each species responds to the experimental apparatus/protocol. Stingless bees are notoriously difficult to work with in a laboratory setting compared to *Apis mellifera*. It is well known that behavioural protocols for bee learning need to be adapted to the specific needs of a species, in order to obtain robust results [11], [12], [16], [46]. It is therefore not impossible that a different set-up, for example under natural conditions in the field using more natural stimuli, might prove successful in demonstrating some degree of arbitrary learning in *Melipona*.

The present study is the first to investigate the acquisition of arbitrary relations in *Melipona rufiventris*, using a symbolic matching-to-sample procedure. Stingless bees have not been as intensively studied as honeybees, and thus our understanding of their biology and cognitive capacity is at an early stage relative to what is known about *Apis mellifera*. The differences in learning performance between the two bee species could be intrinsic and associated with specific foraging and recruitment strategies that have evolved in adaptation to differing foraging environments. However, to provide a conclusive answer, whether and how the cognitive capacities of these bees are driven by their foraging ecology, and whether indeed some bee species are “smarter” than others, detailed studies on the cognitive ecology of a variety of stingless bee species using many different colonies are needed.

## Materials and Methods

### *Apis mellifera* Experiments

The experiments with *Apis mellifera ligustica* were carried out at the Queensland Brain Institute, Australia, in an indoor honeybee flight facility illuminated by natural daylight. Free-flying honeybees from one experimental hive entered the facility through a small window, which was connected to the experimental apparatus. The apparatus consisted of a wooden tunnel (H×W×L = 25×25×130 cm), and three vertical plastic cylinders, (25 cm internal diameter (ID) ×25 cm high: Fig. 1). The cylinders were interconnected via small holes (4 cm ID) to form a Y-shaped maze. The tunnel and the cylinders were covered with UV-transparent Perspex lids. Bees entering the tunnel would fly toward the first cylinder that served to present the sample stimulus. Inside the first cylinder, the bees were then presented with two comparison stimuli (S+ or S–). The correct comparison stimulus (S+) led into another cylinder with a food reward (feeder with 50% w/w sucrose solution), the incorrect comparison stimulus (S–) led to an empty feeder. A paper barrier placed 2 cm behind the holes prevented the bees from seeing the feeders while making their decision. After bees had made their decision, they were released by lifting the lid from the cylinders and returned to the hive. The stimuli were black and white gratings with 3 cm wide bars, printed with a high-resolution laser printer, and colours were pieces of either blue (39N) and yellow (3N) artist-quality cardboard (K+E Stuttgart, Germany). Each stimulus was 14×14 cm in size, with a 4 cm hole in the centre that fitted over the holes in the cylinders.

Individually marked *Apis mellifera* foragers, all experimentally naïve, were initially trained to navigate the Y-maze without stimuli, until they had familiarized themselves with the set-up. During this pre-training phase, the feeder containing sucrose solution was placed first inside the tunnel entrance, then moved step by step through the tunnel, into the first cylinder, and then into the second cylinders alternating position between left and right cylinder (Fig. 1). This procedure, which lasted approximately 2 hours, allowed bees to learn that they had to fly through the holes and into either of the second cylinders to obtain the sugar reward. Bees were considered to be familiar with the set-up, once each individual bee flew straight through the Y-maze and found the feeder at the end without slowing down or turning around.

Then, the visual stimuli were placed on the cylinders to investigate learning of arbitrary relations. One group of bees was trained to relate patterns to colours: when the sample showed the horizontal pattern, the cylinder containing the sucrose was marked with blue; when the sample was the vertical pattern, the cylinder containing the sucrose was marked with yellow. Bees were trained to these stimuli relations in six sessions on six consecutive days. Each session lasted approximately 4–6 hours and consisted of seven training blocks of 30 minutes each. During a training block the sample pattern stimulus and their corresponding colour stimuli were presented alternately, that is bees were first shown one sample stimulus (e.g. horizontal pattern) for 15 minutes, and then the second sample stimulus (vertical pattern) for 15 minutes. During each 15-minute period, the position of the colour comparison stimuli was swapped every 7.5 minutes to prevent positional learning. Typically, a bee returned to the experimental apparatus every 5–8 minutes. This ensured that the bee experienced all stimulus positions at least once during a training block. Bees were individually trained in this way and the experiment was repeated until 20 bees had been trained altogether.

At the end of the six training sessions, after the bees had learnt the arbitrary relations, they were given a test for symmetrical relations. In this test, the stimuli changed their functions: that is, the stimuli previously used as sample were used as comparison stimuli, whereas the stimuli previously used as comparison stimuli were used as sample stimuli. Therefore, the bees were tested for the relations blue-horizontal and yellow-vertical (symmetrical to the relations horizontal-blue and vertical-yellow). A test session consisted of four unrewarded symmetry tests of 15 minutes each; that is, each test sample stimulus was presented twice. Tests were interspersed with 30-minute training blocks with the stimuli in their original function and responses to S+ rewarded to maintain bee motivation. Only the data from the unrewarded tests were used for analysis of the test session.

The entire experiment, i.e. six training sessions over six days for arbitrary relations followed by a test session for symmetric relations, was repeated with a new set of bees, which was trained and tested as described above, but with colours as sample stimuli and patterns as comparison stimuli. The bees had to learn the arbitrary relations blue-horizontal and yellow-vertical, and then they were tested for the symmetrical relations horizontal-blue and vertical-yellow. Again the experiment was repeated until a total of 20 bees were trained and tested.

For analysis, all responses to the comparison stimuli were recorded. The choices from all bees per group were pooled for each block per training session as well as for the four symmetry tests of the test session, and the mean percentage of correct choices per session calculated. Learning performance during training was analyzed using repeated-measures ANOVA with session as within-subject-factor, and performance during the symmetry test was analyzed using a two-tailed one-sample t-test against a reference value of 50%.

### *Melipona rufiventris* Experiments

The experiments with *Melipona rufiventris* bees were carried out at the Universidade Federal de São Carlos, Brazil. A *M. rufiventris* hive was mounted inside the laboratory, which was illuminated by natural daylight, and the experimental apparatus was set up next to the colony to ensure easy access and frequent visits by the bees. The apparatus including UV-transparent Perspex lids and the stimuli were the same as those used for *Apis mellifera* (Fig. 1). The experimental procedure for investigating relational learning in *M. rufiventris* was identical to the one described above for *A. mellifera*. One group of *M. rufiventris* workers, all experimentally naïve was trained to relate patterns to colours, and a second group was trained to relate colours to patterns. As for *Apis*, training was conducted in six sessions of seven 30-minute training blocks each, over 6 consecutive days. Training *Melipona* in complex learning tasks such as matching-to-sample is difficult and time-consuming: it can take many days to ‘entice’ a *Melipona* worker to enter and navigate its way through an experimental apparatus such as the Y-maze used here, and to continually participate in an experiment. Only data from *Melipona* individuals that participated in each consecutive training session were used for analysis. This is in contrast to *Apis* workers, which are easily trained within a couple of hours to participate in practically any behavioural experiment. Therefore, due to the difficulties in training *Melipona* honeybees, only eight *Melipona ruvifentris* workers per group were used. Although fewer bees were used than for *Apis*, the total number of choices per session was in the same range for both species: *Melipona* bees visited the apparatus more frequently as it was set up right next to the hive allowing rapid return visits. For analysis, all responses to the comparison stimuli were recorded. The choices from all bees per group were pooled for each block per training session, and the mean percentage of correct choices per session calculated. Learning performance for arbitrary relations was analyzed using repeated-measures ANOVA.

Due to the outcome of the training sessions investigating relational learning, *M. rufiventris* bees were not tested for symmetrical relations. Instead, they received simple discrimination training with the stimuli used above, to investigate whether they could discriminate the two colours and the two patterns. Discrimination training was conducted straight after the last session of the relational learning experiment. The eight bees from group 1 were trained individually with vertical (S+) versus horizontal (S–) and yellow (S+) versus blue (S–), whereas the other eight bees from group 2 were trained individually with horizontal (S+) versus vertical (S–) and blue (S+) versus yellow (S–). Each bee received eight 15-minute blocks of discrimination training until 100% correct responses in at least two consecutive blocks was reached. The position (left-right) of the stimuli followed a pseudo-random sequence, with the rewarded stimulus being presented on either side for 7.5 minutes during a 15-minute block. Half of the bees in each group were first trained to pattern discrimination and then to colour discrimination; for the other half of the group the sequence was reversed. We did not find any effect of sequence, and thus the choices from all bees per group were pooled for each block irrespective of training sequence, and the percentage of correct choices per block calculated.
